# Impact of Cement Dust Pollution on the Surface of Sound-Absorbing Panels on Their Acoustic Properties

**DOI:** 10.3390/ma13061422

**Published:** 2020-03-20

**Authors:** Artur Nowoświat, Leszek Dulak

**Affiliations:** Faculty of Civil Engineering, Silesian University of Technology, 44–100 Gliwice, Poland; leszek.dulak@polsl.pl

**Keywords:** cement dust, perforated wall cassette, sound absorption coefficient, practical sound absorption coefficient, reverberation chamber

## Abstract

The publication presents a comparison of the sound absorption test results of a perforated wall cassette filled with mineral wool for various degree of cement dust pollution. Cement dust should be understood here as dust created during the production of cement and during the milling and dispatch of finished products. If the partitions in production plants are made of sound-absorbing cassettes or additional sound-absorbing elements made of perforated cassettes are applied, we must know how dust can change sound-absorbing properties of the cassettes. Thus, one has to consider whether the use of sound-absorbing perforated cassettes is appropriate if sound-absorbing parameters change over time due to dust. To determine the impact of dust-covered perforation on sound-absorbing parameters, tests were performed for four variants having different level of pollution. The tests involved ‘clean’ and then dust-covered cassettes, each time increasing the amount of cement dust on the perforations. Sound absorption parameters of the cassettes were tested in the reverberation chamber for individual variants. Test results indicate the loss of sound absorption of the cassettes only when they are heavily polluted. Then the reduction of the single-number sound absorption index *α_w_* is 50%. Using computer simulation, we analyzed how the change of sound-absorbing parameters of the cassettes would influence the change of noise reduction in the production hall. The results of the analysis demonstrate a very effective reduction of noise level of 14 dB by the application of clean cassettes. The reduction value for the dirtiest cassettes was 6 dB.

## 1. Introduction

**Figure 1:**
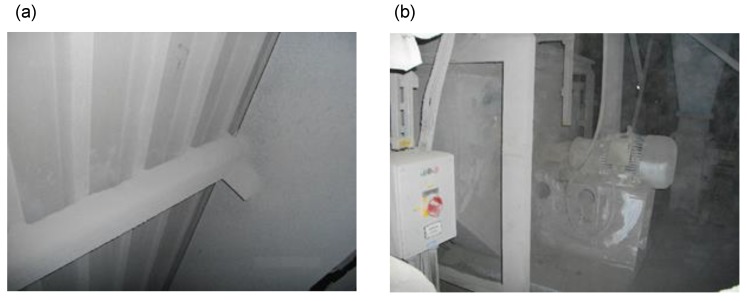
The interior of a factory hall with a visible layer of cement dust: (**a**) on the wall; (**b**) on the fan being the hall’s equipment.

One of the most important roles in terms of room acoustics is played by the phenomenon of reverberation. Reverberation is measured by reverberation time, which is the main criterion in the assessment of the acoustic quality of rooms [1]. To predict reverberation time, various theories [2] are applied, which comprise the geometric theory [3], wave theory [4], or the theory based on the least squares and subsequent approximations [5]. The reverberation time in rooms depends on acoustic absorption, which, in turn, depends mainly on the sound absorption of materials used for their construction. For this reason, perforated cassettes with mineral wool filling are often used as building envelopes [6]. One of the reasons to decrease reverberation time in rooms is the need to reduce reverberation noise. This is important not only in public utility buildings but also in industrial facilities [7]. Excessive noise has a negative impact on the health of people exposed to it [8]. According to Oishi and Schacht [9], according to the WHO report, it was estimated that 10% of the world’s population is exposed to high levels of sound pressure. One of the negative effects of noise on human health is hearing loss. It is also referred to as occupational hearing loss [10], and it accounts for almost one third of all occupational diseases in Europe [11]. As already mentioned, excessive noise can be reduced by using appropriate sound-absorbing materials, which include natural fibers [12], or polyurethane foam [13]. For mechanical protection of the fragile sound-absorbing layer, a perforated metal cladding is applied on the side of the wall exposed to noise [14]. Nevertheless, we must remember that in a production hall, depending on the production process being carried out, sound-absorbing panels may be considerably dirty. One of the most dust-generation production processes involves cement production. Figure 1a,b presents photos of an exemplary factory hall where, as a result of pollution with cement dust, the partitions and elements of the hall equipment have become significantly dust-polluted.

In view of the above, the authors present the results of research studies on the impact of the extent of pollution of perforated sound-absorbing panels with cement dust on selected acoustic parameters. The tested acoustic parameters comprised sound absorption coefficient α_s_, determined for 1/3 octave bands, the practical sound absorption coefficient α_p_ specified for octave bands and the single-number sound absorption index α_w_. Those values are entry for the prediction of the expected noise level reductions.

## 2. Methodology

### 2.1. Sound-Absorbing Material

**Figure 2:**
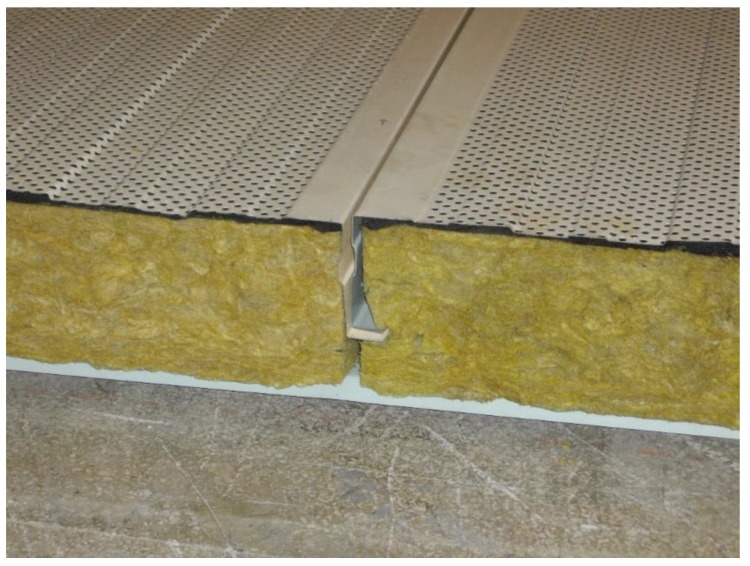
Fragment of the light curtain wall being the sample subjected to acoustic tests. Details of the test set-up on the floor of the reverberation room.

The structure of the tested wall is presented in Figure 2. The cassette sheet 0.65 mm being the top layer of the tested sample had the perforation in the form of round holes of the diameter of 6.0 mm with a spacing of 11.24 mm. The degree of perforation of the pattern was 25.1%, while the actual degree of perforation of the cassette face was 18.2%. The sound-absorbing layer consisted of 140 mm-thick rock mineral wool 55 kg/m^3^. The bottom layer was made of the 0.63 mm-thick trapezoidal metal sheet TRB-35/1035.

In order to simulate the pollution of the perforation with cement dust, analogous to that occurring on the walls in cement plant rooms, a sample was tested in four variants:

- Variant A—wall without dirt;
- Variant B—“dirty” wall, 5.0 kg of cement dust/12 m^2^ of the sample;
- Variant C—“dirty” wall, 19.5 kg of cement dust/12 m^2^ of the sample;
- Variant D—“dirty” wall, 24.5 kg of cement dust/12 m^2^ of the sample, sprayed with water (6 L/12 m^2^), tested after 72 h from the moment of spraying (spraying with water was intended to simulate the situation in which the cement dust covering the panels becomes damp as in real conditions).

**Figure 3:**
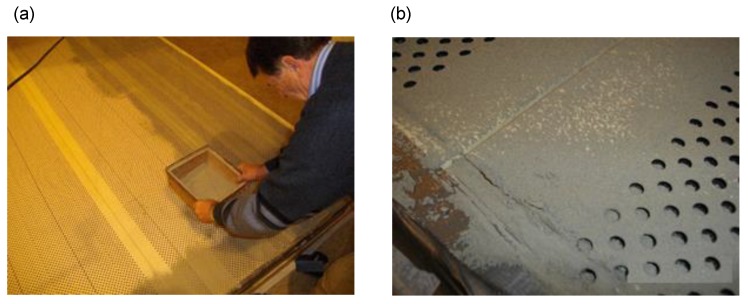
(**a**) Polluting of the sample with a sieve, (**b**) the sample after being sprinkled with cement dust in the amount of 5.0 kg/12 m^2.^

The samples were being polluted with cement dust collected from a cement plant, where it had accumulated on the elements of the production line and on partitions. The dusting process of samples for each variant was carried out in a different way. For variant B, the pollution was made by sprinkling the sample with cement dust, using a sieve as presented in Figure 3a,b.

**Figure 4:**
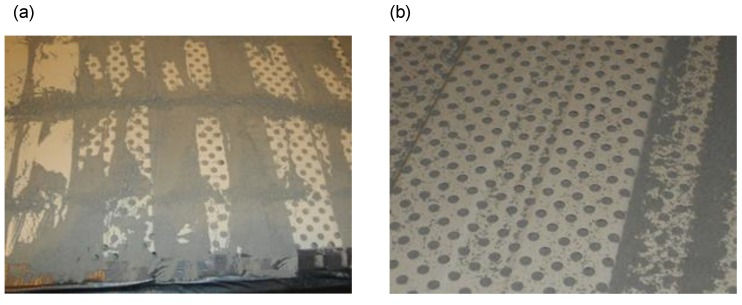
(**a**) Polluting the sample. (**b**) The sample after being sprinkled with cement dust in the amount of 19.5 kg/12 m^2^.

For variant C, the pollution was made by repeatedly spreading cement dust on the wall surface with a broom. The excess cement dust that did not fill up the perforation, and the space between the metal sheet and mineral wool was removed manually. This process is presented in the photographs shown in Figure 4a,b.

**Figure 5:**
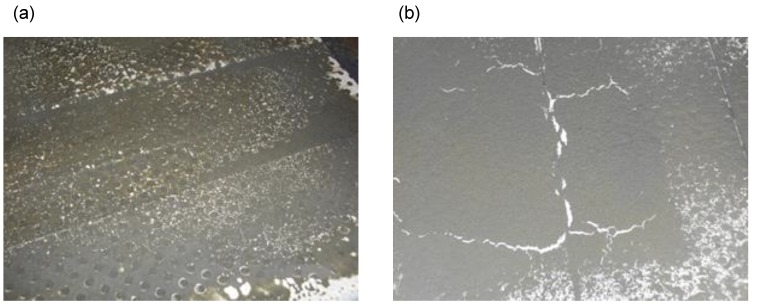
(**a**) Polluting the sample; (**b**) The solidified sample after the application of dust-pollution of 24.5 kg/12 m^2^ and water sprinkling of 6 L/12 m^2^.

For variant D, the pollution was made by dusting the sample C in the amount of 19.5 kg/12 m^2^ using a sieve with an additional amount of cement dust of 5.0 kg/12 m^2^, obtaining a total dust-pollution of 24.5 kg/12 m^2^. Then the whole was sprinkled with water, using sprayers in the amount of 6 L/12 m^2^, so that a “crust” could develop on the surface of the sheet as presented on the photographs in Figure 5a,b.

### 2.2. Measurement of Sound Absorption Coefficient

The tests were carried out using a measuring system whose components meet the metrological requirements for instruments of accuracy class 1.

The transmitting part of the system consisted of the following elements: an omnidirectional sound source, a pink noise generator with an amplifier. The reception part of the system included the following elements: a four-channel sound level meter SVAN 958 (Svantek, Warsaw, Poland), a 1/2” microphone SV22 (Svantek, Warsaw, Poland), a 1/2” microphone preamplifier SV12L (Svantk, Warsaw, Poland), an acoustic calibrator SV03A (Svantek, Warsaw, Poland), PC with software. The devices had valid certificates of authentication and verification.

**Figure 6:**
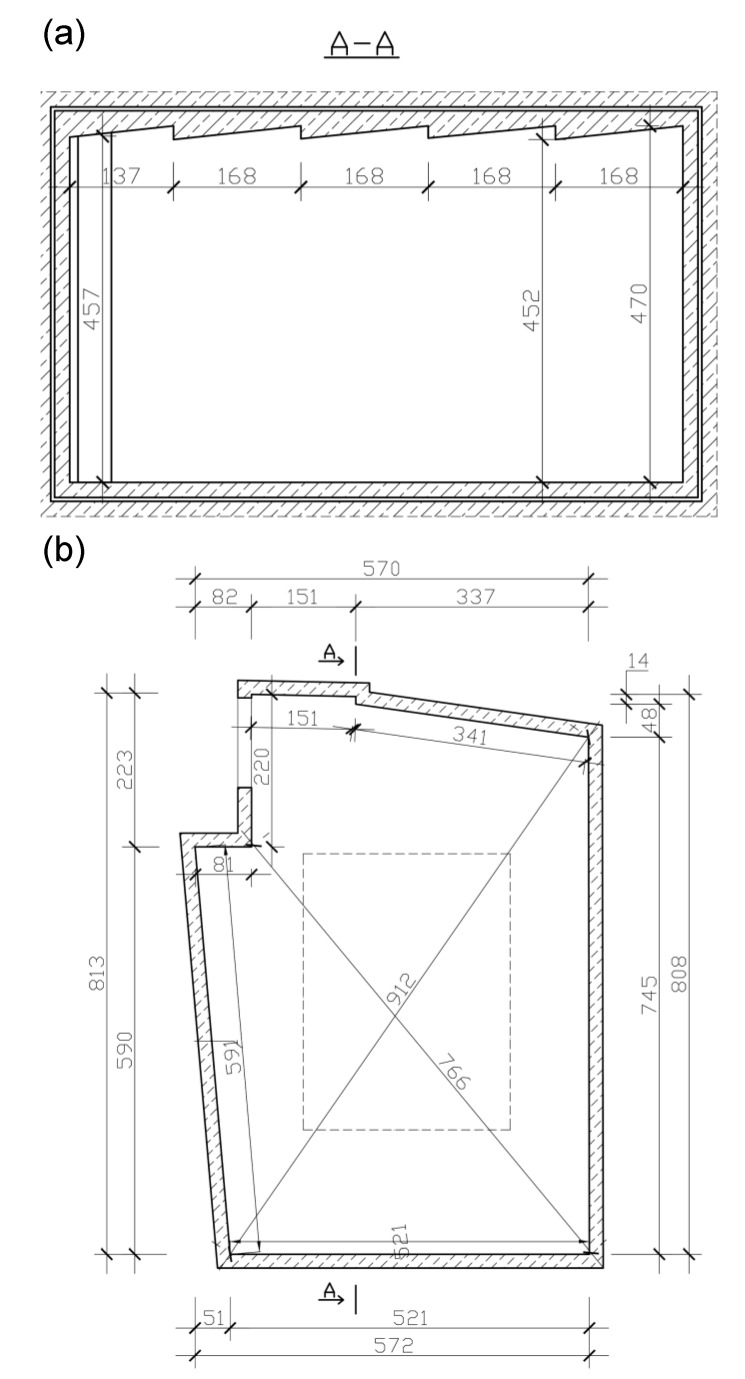
Reverberation chamber. (**a**) Cross-section of the reverberation chamber. (**b**) Projection of the reverberation chamber.

The study of sound absorption of the material was carried out in a reverberation chamber located in the Laboratory of the Faculty of Civil Engineering of the Silesian University of Technology in Gliwice [15]. The dimensions of the chamber of the volume of V = 192.7 m^3^ are presented in Figure 6a,b.

**Figure 7:**
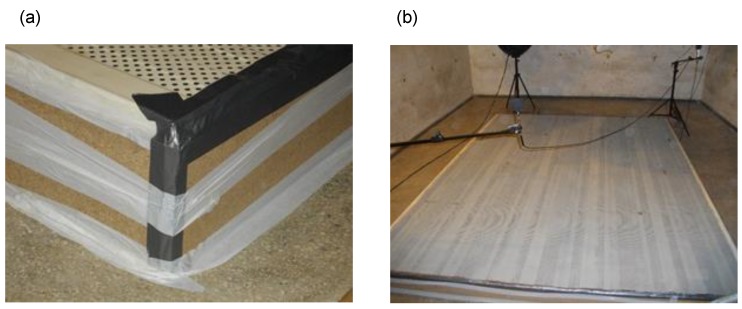
View of the test sample during the measurements of sound absorption coefficient. (**a**) Protection of the periphery of the test sample in order to minimize sound absorption by the edge of the sample. (**b**) View of the sample placed on the floor of the reverberation chamber.

The wall sample was placed on the floor of the reverberation chamber, in line with the recommendations for the installation method according to the standard [16], with the perforation directed upwards. The sample was placed in the reverberation chamber 24 h before the first measurements were made. The tested sample had the dimensions: 400.0 × 300.0 × 18.0 cm. The rim was shielded with a frame made of chipboards 10 mm thick and taped in the corners to eliminate sound absorption by that element of the sample. The photographs presented in Figure 7 show the sample during the tests in the reverberation chamber. Figure 7a presents the protection of the sample rim against sound absorption, while Figure 7b presents the test sample.

The tests were carried out using the intermittent noise method in line with the guidelines contained in the said standard [16].

The measurements for each of the samples were made for six microphone locations and two sound source locations, which gave a number of spatially independently measured decay curves equal to 12. For each of the 12 microphone/loudspeaker positions, in order to reduce measurement uncertainty caused by statistical deviations, 6 repetitions were carried out. For each of the 72 sound decay curves, the reverberation time was determined. The final result was the arithmetic average. Similarly, the tests were carried out for an empty chamber (without the sample). The measurements of the reverberation time of the empty chamber were made immediately after dismantling the test sample.

### 2.3. Determination of Sound Absorption Coefficient

Based on the measurements of reverberation time, equivalent sound absorption fields *A*_1_ (empty room) and *A*_2_ (room with sound-absorbing material) are determined in square meters:

(1) $$A_{1}=\frac{55.3V}{c_{1}T_{1}}-4m_{1}V;A_{2}=\frac{55.3V}{c_{2}T_{2}}-4m_{2}V$$

As a result, we can determine the equivalent sound absorption field of the tested sample:

(2) $$A_{T}=A_{2}-A_{1}=\frac{V}{c_{2}T_{2}}\left[55.3\left(\frac{1}{c_{2}T_{2}}-\frac{1}{c_{1}T_{1}}\right)-4\left(m_{2}-m_{1}\right)\right]$$

By repeating this procedure for each one-third octave frequency band f, we determine sound absorption coefficient for each of such bands:

(3) $$\alpha_{s}=\frac{A_{T}}{S}$$

*V*—volume, in cubic meters, of the empty reverberation room, m^3^

*A*_1_—equivalent sound absorption area of the empty reverberation room, m^2^

*A*_2_—equivalent sound absorption area of the reverberation room containing the test specimen, m^2^,

*c*_1_, *c*_2_—propagation speed of sound in the air, in m/a, calculated using the formula c=331+0,6t with t—air temperature in degrees Celsius for temperatures in the range of 15 °C to 30 °C

*T*_1_— reverberation time, in seconds, of the empty reverberation room,

*T*_2_—reverberation time, in seconds, of the reverberation room after the test specimen has been introduced,

*m*_1_, *m*_2_—power attenuation coefficient, in reciprocal meters, calculated according to [17]

*A*_T_—equivalent sound absorption area of the test specimen in square meters

*S*—area, in square meters, covered by the test specimen

*α*_s_—sound absorption coefficient.

Then, for each i-th octave band, the practical sound absorption coefficient α_pi_ is calculated as the arithmetic mean. The said coefficient is the arithmetic mean of the sound absorption coefficients α_s_ for 1/3 octave bands contained in the analyzed octave.

Then we determine the coefficient α_w_. The said coefficient is a single-number quantity, independent of frequency, whose value is equal to the value of the reference curve for 500 Hz, after shifting in the way specified by the standard [18].

## 3. Results and Discussion

### 3.1. Results of Laboratory Tests

**Figure 8:**
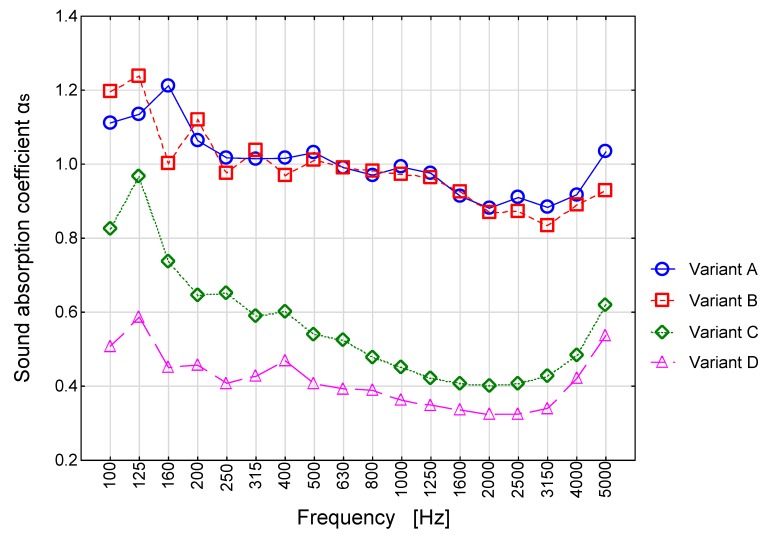
Sound absorption coefficient as a function of frequency for the four tested variants.

The structure of the tested wall is presented in Figure 2. The cassette sheet being the upper layer of the tested sample was perforated. The method for determining sound absorption coefficient was presented in Section 2.3, and the determined sound absorption coefficients were presented in Figure 8.

We can observe an unexpected effect for variants A and B on the graphs in Figure 8, where for low frequencies, the value of sound absorption coefficient *α*_si_ higher than 1 was obtained, whereas in terms of the standards the said value is contained within the interval 〈0,1〉. These values are the exact measured values including the “area or boundary effect”. Such a situation is quite common for measurements of strongly absorbing samples in reverberation chamber conditions. As written for instance by Everest [19], scattering of sound at the edges of the research sample brings about the situation as if the effective sample area increases so leading to Sabine absorption values higher than 1.

As it can be easily observed from the graph in Figure 8, the impact of the amount of dust and water on the sound absorption coefficient of the sound-absorbing panel is significant. In principle, we can state that light pollution of the sound-absorbing panel with cement dust applied with a sieve in the amount of 5.0 kg/12 m^2^ does not impair the sound-absorbing properties. On the other hand, the use of almost four times more cement dust (Variant C) results in a significant reduction of the sound-absorbing parameters of the panel. The above effect is noticeable for all 1/3 octave frequency bands. An additional increase of the amount of cement dust and, above all, the use of water in the amount of 6 L/12 m^2^ resulted in a further significant reduction of the sound-absorbing parameters of the panel.

The measurement uncertainty described by the Formula (4) was determined on the basis of ISO 354: 2003 [16]:

(4) $$\delta_{95}\left(\alpha \right)≅\frac{1.96\varepsilon \left(\alpha \right)}{\sqrt{N}}$$

For variant A, the measurement uncertainty ranged from 0.003 to 0.006 depending on the frequency band, for variant B the range was 0.003–0.005, for variant C 0.002–0.004 and for variant D 0.001–0.004.

Using the determined values of practical sound absorption coefficient α_p_, the single-number sound absorption index α_w_ was determined.

Variant A—*α*_w_ = 1.00 (L),

Variant B—*α*_w_ = 0.95 (L),

Variant C—*α*_w_ = 0.45 (L),

Variant D—*α*_w_ = 0.40 (L).

Where (L) stands for the so-called shape determinant. In line with the standard [18], the shape determinant L, M, H means that the value of the coefficient *α*_pi_ exceeds by 0.25 or more the shifted reference curve in different frequency bands. In the case of the results presented above, the shape determinant L means that in the low frequency band the analyzed material has higher absorption properties than the value of the index *α*_w_ would indicate.

### 3.2. Results of Computer Simulations

**Figure 9:**
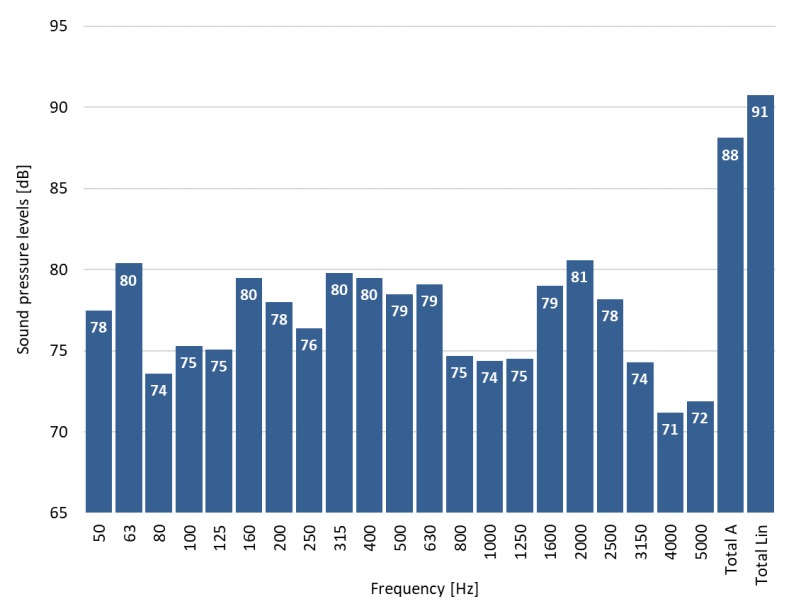
Averaged measurement results of the immission of sound pressure level at the distance of 1.0 m from the fan in 1/3 octave frequency bands and the value of total A and total lin.

The decrease of the sound-absorbing parameters of sound-absorbing cassettes observed in the tests due to contamination with cement dust will in practice lower their effectiveness in reducing noise. The question is how much noise will increase in the room where sound-absorbing cassettes are polluted with dust. To answer the above question, we attempted to use computer simulations. For learning purposes, the authors analyzed an exemplary room with simple geometry similar to the real dimensions of production halls found in the industry. The simulations involved noise immission by an industrial fan. The acoustic parameters of the fan were adopted on the basis of field measurements carried out in the cement plant production hall. Based on the measurements, sound pressure level was determined at the distance of 1.0 m from the fan (the measuring distance 1.0 m was required by the computer program). Figure 9 shows the averaged measurement results of the immission of the sound pressure level from the fan in 1/3 octave frequency bands and the value of total A and total lin.

**Figure 10:**
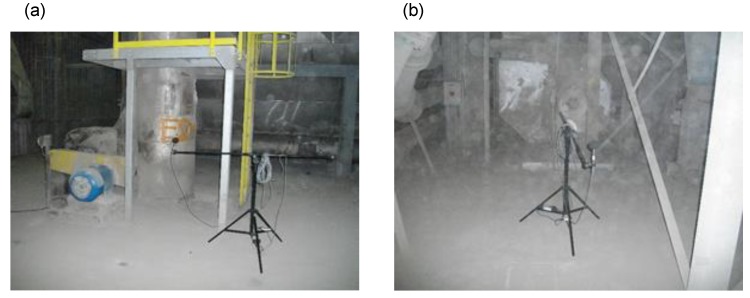
Immission measurement of sound pressure level from the fan in a cement plant production hall. (**a**) View of sound analyzer and the fan in the background. (**b**) Apart from the sound analyzer, the photo shows cement dust floating in the air indicating significant dustiness.

The above data were implemented to the computer model as noise source data. Figure 10a shows the view of the fan and sound analyzer during the measurement of sound pressure level. Figure 10b also involves the measurement of noise from the fan. Moreover, we can observe on it the dust associated with the technological process carried out in the cement plant.

**Figure 11:**
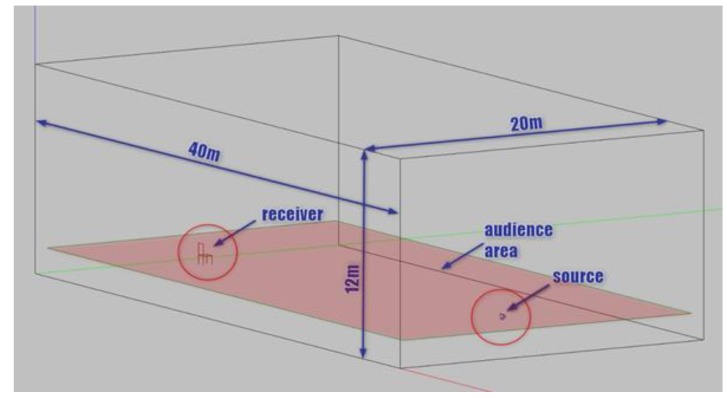
Geometric model that was used to calculate the immission of noise level using the program EASE, version 4.4.61.

Figure 11 presents the geometric model that was used to determine the impact of the application of sound-absorbing cassettes on the reduction of noise level in a production hall depending on the extent of cement dust contaminating them.

**Figure 12:**
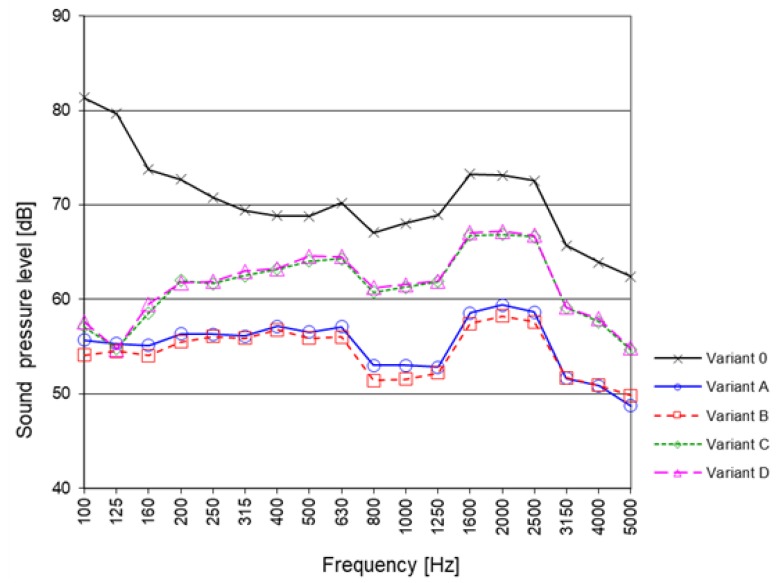
Calculation Results of sound pressure level from the fan at the reception point in the 1/3 octave frequency bands for variants 0, A, B, C and D.

**Figure 13:**
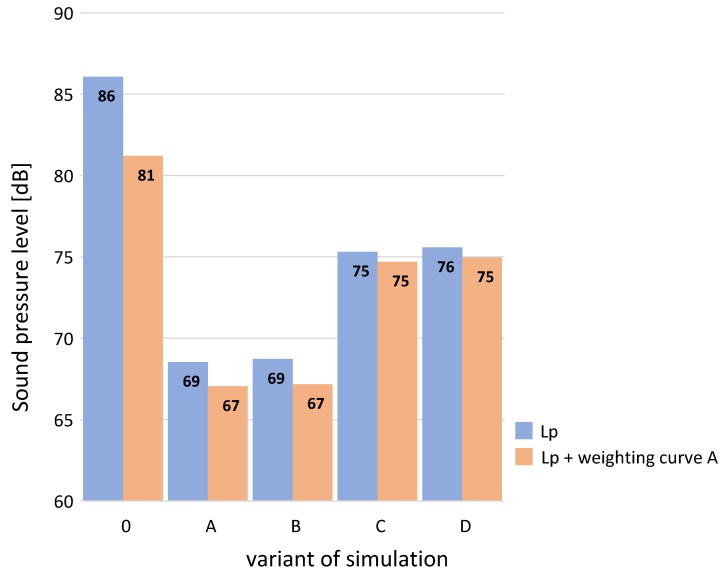
Calculation results for sound pressure level from the fan at the reception point in the 1/3 octave frequency bands for variants 0, A, B, C and D.

The simulation calculations of noise level immission were carried out using the Aura module of the computer program EASE, version 4.4.61. The AURA module—Analysis Utility for Room Acoustics—is an acoustical analysis tool. Based on the CAESAR algorithms developed by Aachen University (RWTH), AURA allows to calculate key room acoustical parameters. AURA uses both a deterministic Image Model (Cone Tracing) and Stochastic Ray Tracing methods. In order to carry out the above task, simulations were performed for the room with a concrete floor and walls made of cassettes without perforation (variant 0). Then sound-absorbing cassettes (Budmat, Rogozino, Poland) were used on the walls and the ceiling, whereof test results are presented in Section 3.1, respectively in variants A, B, C and D. Figure 12 presents the calculation results of sound pressure level from the fan at the reception point in the 1/3 octave frequency bands for subsequent variants. Figure 13 presents the same noise level results for the realized calculation variants, but in the form of total value in dB and in dB (A) after the correction with weighting curve A in line with ISO 61672. 

The obtained results demonstrate a significant potential to reduce noise level in the room through the use of sound-absorbing cassettes on the walls and ceiling. In the case of variants A and B, the noise reduction L_pA_ is by 14 dB (81 dB → 67 dB). Thus, the results demonstrate that the cassettes polluted with cement dust have only a small level of efficiency (variant B - 5 kg /12 m^2^) as compared to that demonstrated by clean screens (variant A). For variants C and D (cassettes significantly polluted with dust) the reduction was 6 dB (81 dB → 75 dB). The above value may seem small in terms of the results presented in the 1/3 octave bands in Figure 12. It demonstrates a significant reduction of noise for samples C and D in the low frequency range (for 100 Hz it is approx. 24 dB). Nevertheless, the said reduction does not translate into the total value expressed in dBA (i.e., after the correction, as for the equal loudness characteristics for low sound levels). More promising is the reduction expressed in total lin (or total Z). In this case, the reduction is as much as 11 dB (variant C) and 10 dB (variant D). For the case of a fan noise, which reaches a significant level, it seems to be more appropriate.

**Figure 14:**
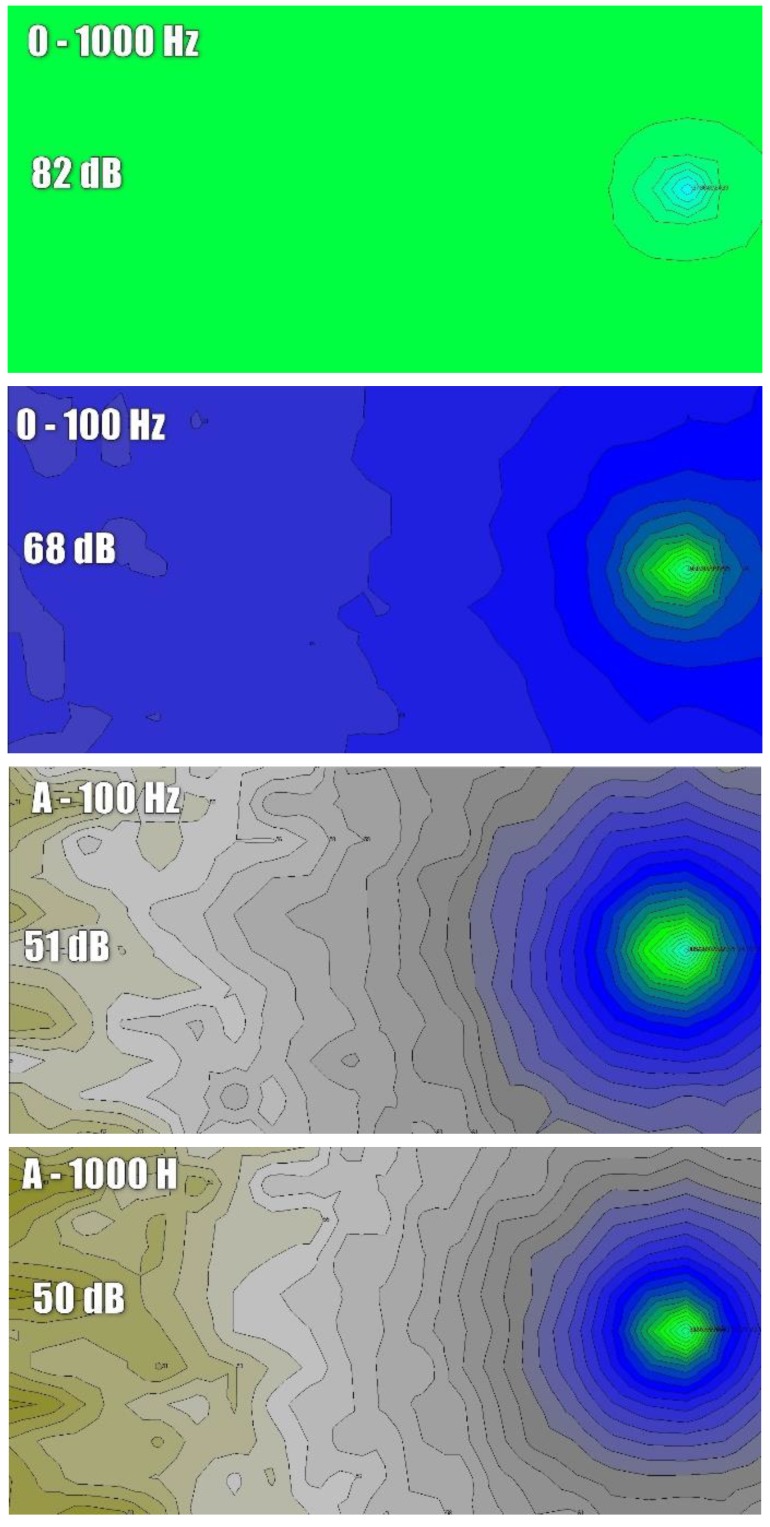

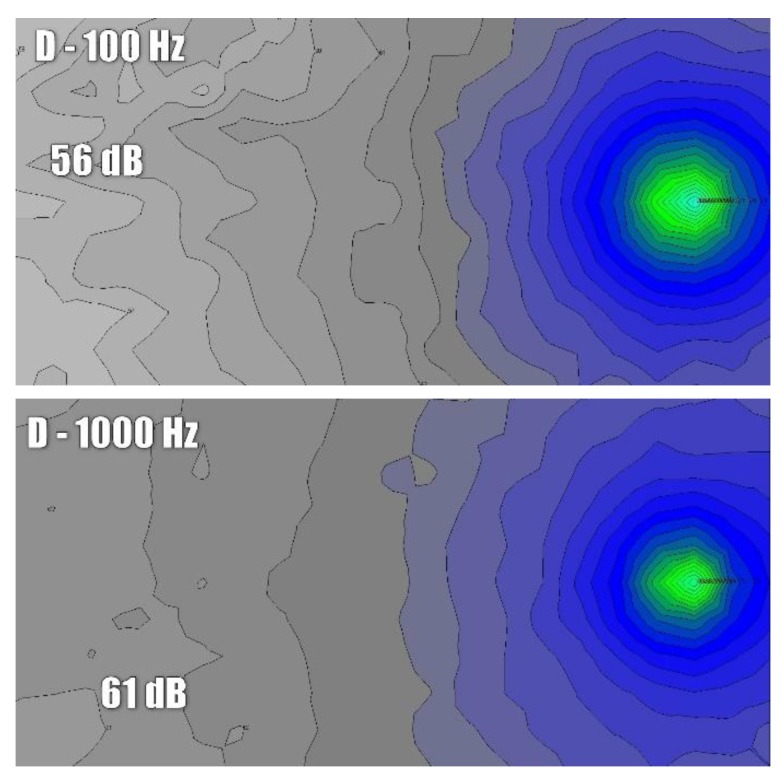
Calculation results of sound pressure level in the audience area in the form of noise maps for variants 0, A and D for the selected 1/3 octave bands of the central frequency of 100 and 1000 Hz.

The observations regarding good noise reduction at low frequencies, regardless of the pollution level of the cassettes, are confirmed by the results presented in Figure 14. It presents the calculation results of sound pressure level in the audience area in the form of noise maps for variants 0, A and D. We selected the 1/3 octave bands with the center frequency of 100 and 1000 Hz to demonstrate the difference in noise reduction at low and high frequencies. Based on the compiled results, we can observe that for variant 0 (production hall without sound-absorbing cassettes) for the frequency of 100 Hz, sound pressure level throughout the entire audience area practically does not change and does not drop below 82 dB (green color dominates). The situation for higher frequencies looks slightly better. For the selected graph for 1000 Hz, we can see that with the distance from the fan green color gives way to blue. At the opposite end of the hall, sound pressure level reaches 68 dB. For variant A, the difference between the acoustic map for 100 and 1000 Hz is small. This confirms earlier observations that clean sound-absorbing cassettes effectively reduce noise in a wide frequency range. For both selected frequencies the reduction is to approx. 50 dB. In the case of very polluted cassettes (variant D), noise reduction looks better for low frequencies. Hence, in the figure representing the frequency of 100 Hz, we observe a decrease to 56 dB, while for the frequency of 1000 Hz, the decrease is slightly lower, and the level of sound pressure reaches 61 dB.

## 4. Conclusions

The shortage of information on how dusting of sound-absorbing cassettes affects their acoustic parameters was the reason why the authors decided to undertake research in this area. The measurements carried out for four variants of dust pollution degree of the cassettes demonstrate a close relationship between the amount of dust deposited on the cassettes and sound absorption coefficient. When the samples are sprinkled with cement dust, the reduction of their sound-absorbing parameters is small and amounts only to 5% (the change of the α_w_ index from 1.00 for variant A to 0.95 for variant B). However, when the sound-absorbing cassettes are polluted with the maximum amount of cement dust which could be introduced under the cassette perforation (24.5 kg/12 m^2^), and additionally moisture is applied, the reduction of the single-number sound absorption index α_w_ amounts to as much as 60%. There was a change in the α_w_ index from 1.00 for the “clean” cassette to 0.40 for the cassette in Variant D. It should be noted that the measurement procedures used in the article based on [16] are subject to uncertainty of results, which can be verified in particular cases by means of air flow measurements [20]. In the work we attempted to verify it by using appropriate software. The results of the computer simulation carried out indicate that there is a significant risk of noise increase if sound-absorbing cassettes are polluted with a significant amount of cement dust. As compared to the 14 dB-reduction achieved for the exemplary room through the use of clean cassettes, the polluted cassettes (variants C and D) yielded the reduction only by 6 dB. According to the authors, the obtained results do not disqualify the use of sound-absorbing cassettes in rooms with high dustiness. In view of the obtained results, we can realistically determine the achievable noise reduction in time perspective when the panels are dusted. Based on the obtained results, we can state that when designing facilities in which dust pollution is significant, this fact should be taken into account. It seems appropriate to choose the number of sound-absorbing elements with some surplus so that over time the reduction of their sound-absorbing properties would not contribute to exceeding the permissible noise levels in the work environment. For future research, it would also be interesting to quantify the impact of dust deposition on nominally “sound reflecting” materials. The increase of sound absorption and the complimentary decrease of sound reflection can be measured in the same way as described.
